# Robot-Assisted Versus Open Nephrectomy with Inferior Vena Cava Thrombectomy in Renal Cell Carcinoma: Current Evidence and Surgical Trends

**DOI:** 10.3390/cancers18142251

**Published:** 2026-07-14

**Authors:** Zuzanna Korbecka, Beata Jabłońska, Robert Król

**Affiliations:** 1 Department of General, Vascular and Transplant Surgery, Medical University of Silesia, St. Francuska 20-24, 40-064 Katowice, Poland; robertk@hot.pl; 2 Department of Digestive Tract Surgery, Medical University of Silesia, St. Medyków 14, 40-753 Katowice, Poland; bjablonska@sum.edu.pl

**Keywords:** renal cell carcinoma, inferior vena cava, tumor thrombus, robotic surgery, radical nephrectomy, thrombectomy, surgical oncology, systematic review

## Abstract

Background/Objectives: Renal cell carcinoma (RCC) with inferior vena cava tumor thrombus (IVCTT) is a rare and surgically complex condition traditionally managed with open nephrectomy and thrombectomy. Robot-assisted approaches are increasingly used in selected patients, but their comparative perioperative and oncological value remains uncertain. This systematic review aimed to summarize current evidence comparing robot-assisted and open nephrectomy with IVC thrombectomy in RCC. Methods: A systematic search of PubMed, Scopus, Web of Science, and Cochrane Library was performed according to PRISMA guidelines. Original clinical studies reporting perioperative or oncological outcomes of nephrectomy with IVC thrombectomy were included. Owing to substantial heterogeneity, a qualitative synthesis was performed. Results: Thirteen studies were included. Robot-assisted surgery was associated with lower estimated blood loss, reduced transfusion rates, and shorter hospital stay, although operative time was generally longer. Complication rates and oncological outcomes were broadly comparable between approaches. Robotic cohorts were typically highly selected and included mainly patients with lower-level thrombi, limited tumor burden, and procedures performed in high-volume centers. Conclusions: Robot-assisted nephrectomy with IVC thrombectomy appears feasible and safe in carefully selected patients and may improve perioperative outcomes without compromising oncological efficacy. However, open surgery remains essential for complex high-level thrombi. Tumor thrombus level, tumor burden, and institutional expertise should guide surgical approach selection.

## Simple Summary

Renal cell carcinoma can extend into the inferior vena cava, creating a complex surgical condition that has traditionally required open surgery. This review summarizes current evidence comparing robot-assisted and open nephrectomy with inferior vena cava thrombectomy. Robot-assisted surgery appears to reduce blood loss, transfusion rates, and hospital stay in carefully selected patients, particularly those with lower-level tumor thrombi. However, oncological outcomes seem comparable between approaches and are mainly influenced by tumor-related factors such as thrombus level, tumor volume, and vascular invasion. Open surgery remains essential for advanced thrombi and complex vascular involvement.

## 1. Introduction

Renal cell carcinoma (RCC) with inferior vena cava tumor thrombus (IVCTT) represents a rare but clinically challenging entity, occurring in approximately 4–10% of patients [1,2,3]. Surgical resection with radical nephrectomy and thrombectomy remains the standard of care and offers the only potential for cure [1,4,5,6,7,8,9,10,11].

Traditionally, these procedures have been performed using an open approach because of their technical complexity and the need for reliable vascular control [4,10,12,13,14,15,16,17,18]. However, advances in minimally invasive surgery, particularly robot-assisted techniques, have expanded the surgical armamentarium and enabled selected cases to be managed using a robotic approach [19,20,21,22,23,24,25,26,27].

Despite increasing adoption of robot-assisted surgery, the available evidence remains limited and largely based on retrospective studies. Moreover, the relative contribution of surgical approach compared with tumor-related factors, such as thrombus level and tumor burden, remains incompletely understood [9,19,20].

The aim of this study was to systematically review current evidence on robot-assisted versus open nephrectomy with IVC thrombectomy, focusing on perioperative outcomes, oncological results, and determinants of surgical complexity.

## 2. Surgical Techniques and Technical Considerations

Surgical management of RCC with IVCTT is technically demanding and requires a tailored approach based on thrombus level, tumor characteristics, and institutional expertise. Both open and robot-assisted approaches share fundamental oncological principles, including complete tumor excision and safe vascular control, although their technical execution differs.

### 2.1. Surgical Access

Open radical nephrectomy with IVC thrombectomy is typically performed via a transperitoneal approach using a midline laparotomy, bilateral subcostal, or chevron incision, allowing wide exposure of the kidney, great vessels, and surrounding structures. In selected cases, particularly with higher-level thrombi, extended exposure including thoracoabdominal access or liver mobilization may be required [4,10,12,14,16,17].

Robot-assisted procedures are most commonly performed via a transperitoneal approach, which provides adequate working space and optimal visualization of the IVC. In selected cases, particularly for left-sided tumors, a staged or repositioning strategy may be required to facilitate both caval thrombectomy and nephrectomy [21,22,23,27]. A retroperitoneal robotic approach has been described but remains limited to highly selected cases with lower-level thrombi [19,20,28].

### 2.2. Control of the Inferior Vena Cava

Adequate vascular control is a critical step in both open and robotic surgery. In open procedures, direct manual exposure allows sequential clamping of the infrarenal IVC, contralateral renal vein, and suprarenal IVC before cavotomy, ensuring hemodynamic stability and minimizing the risk of embolization [4,10,12,14,16,17].

In robotic surgery, vascular control is achieved using vessel loops, Rummel tourniquets, or laparoscopic bulldog clamps. Circumferential dissection of the IVC is performed to allow proximal and distal control before venotomy. Depending on thrombus extent, either complete cross-clamping or lateral clamping may be employed to maintain a bloodless surgical field [19,20,21,22,23,27].

### 2.3. Key Operative Steps

The main operative steps are consistent across approaches. Early ligation of the renal artery is commonly performed to reduce venous congestion and facilitate thrombus mobilization. Subsequent dissection of the IVC allows identification of the proximal and distal limits of the thrombus.

Following vascular control, cavotomy is performed and the IVCTT is carefully extracted. In cases of non-adherent thrombus, simple extraction may be sufficient, whereas more advanced cases may require partial resection of the IVC wall and reconstruction. Caval closure is typically achieved using a running suture, with attention to maintaining luminal patency and minimizing stenosis. For higher-level thrombi, additional maneuvers such as liver mobilization, the Pringle maneuver, or cardiopulmonary bypass may be necessary, particularly in open surgery [21,22,23,26].

### 2.4. Robotic Platforms

Most reported robot-assisted procedures have been performed using the da Vinci Surgical System, which provides high-definition three-dimensional visualization and articulated instruments that facilitate precise dissection and suturing. Emerging robotic systems, such as the Versius Surgical System, are increasingly being adopted; however, their role in complex vascular procedures such as IVC thrombectomy remains to be fully established.

### 2.5. Technical Considerations and Learning Curve

Robot-assisted thrombectomy requires advanced expertise in both robotic surgery and vascular control techniques. Operative time and perioperative outcomes are influenced by the surgical learning curve, with improvements observed after increasing case volume. Additionally, longer IVC clamping times have been reported in robotic procedures; however, current evidence suggests that this does not significantly affect renal function or thromboembolic risk [25,26].

## 3. Methods

### 3.1. Search Strategy

A systematic literature search was performed in PubMed, Scopus, Web of Science, and Cochrane Library databases. The search strategy included controlled vocabulary and free-text terms related to RCC, IVCTT, nephrectomy, and robot-assisted surgery.

The search was limited to English-language studies involving human subjects; non-English publications were excluded during the screening process. No restriction on publication year was applied.

The complete database-specific search strategies are provided in Supplementary Table S2 to improve transparency and reproducibility of the review process.

### 3.2. Study Selection

This systematic review was reported in accordance with the PRISMA 2020 statement. The completed PRISMA 2020 checklist is provided as Supplementary Table S1 [29,30]. The review was not prospectively registered in PROSPERO, as the study had progressed beyond the data extraction stage at the time of registration consideration.

A total of 663 records were identified. After removal of duplicates, 300 records remained for title and abstract screening. Of these, 23 full-text articles were assessed, and 13 studies met the inclusion criteria.

### 3.3. Inclusion and Exclusion Criteria

Studies were included if they evaluated RCC with IVCTT, reported perioperative and/or oncological outcomes, included robotic and/or open surgical approaches, and were original clinical studies.

Studies were excluded if they were reviews, editorials, conference abstracts, case reports, lacked original patient data, or did not specifically address IVCTT.

### 3.4. Data Extraction

Data extracted included study characteristics, surgical approach, tumor thrombus level, perioperative outcomes (operative time, blood loss, transfusion rate, length of stay, and complications), and oncological outcomes.

### 3.5. Outcomes

Primary outcomes were perioperative parameters. Secondary outcomes included complications and survival outcomes, including overall survival (OS), cancer-specific survival (CSS), and progression-free survival.

### 3.6. Risk of Bias Assessment

Risk of bias was assessed using the Newcastle-Ottawa Scale (NOS) for nonrandomized studies [31]. Studies scoring 7–9 points were considered high quality, 5–6 points moderate quality, and fewer than 5 points low quality.

The risk of bias assessment using the Newcastle-Ottawa Scale is summarized in Table 1.

Most included studies were retrospective observational cohorts. Comparative multi-institutional studies generally demonstrated higher methodological quality due to better cohort comparability and more comprehensive outcome reporting. Smaller robotic case series were associated with moderate risk of bias, primarily related to limited sample size, retrospective design, and shorter follow-up duration.

### 3.7. Data Synthesis

**Figure 1:**
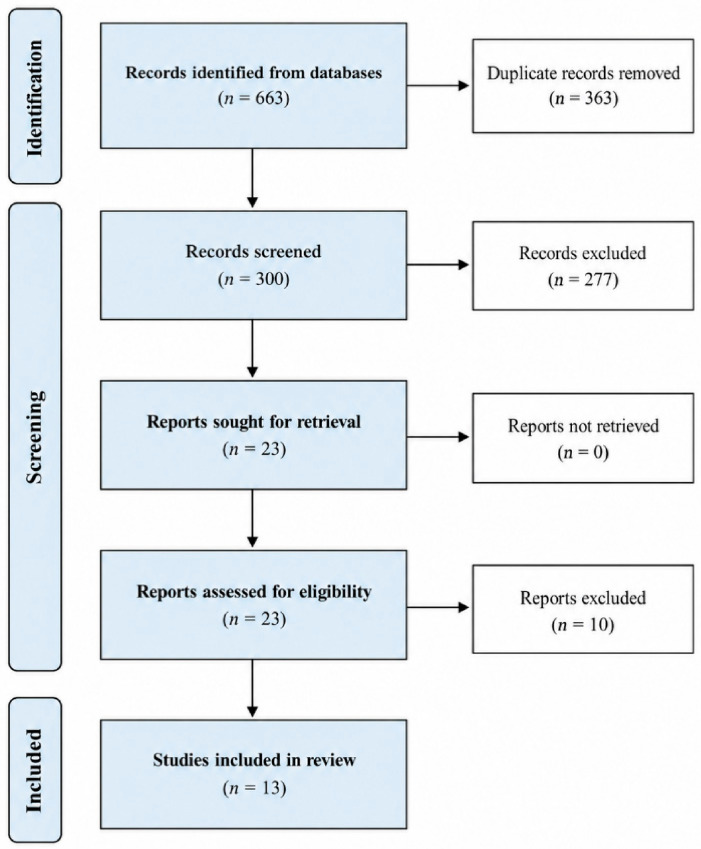
PRISMA flow diagram of study selection.

Due to substantial heterogeneity in study design, patient populations, and outcome reporting, a qualitative synthesis was performed [41]. Figure 1 presents the PRISMA flow diagram of study selection.

## 4. Results

### 4.1. Characteristics of Included Studies

Thirteen studies were included, comprising comparative analyses, robotic case series, and observational cohorts. Most studies were retrospective and conducted in high-volume centers [33,37,40]. Sample sizes ranged from small robotic series (*n* = 5–32) to large multi-institutional cohorts exceeding 300 patients [33,38,39].

Table 2 summarizes the baseline characteristics of the included studies and patient populations.

IVCTT levels ranged from Mayo I to IV. However, robotic surgery was predominantly applied in patients with lower-level thrombi (Mayo I–II), whereas open surgery encompassed the full spectrum of thrombus levels, including more advanced cases (Mayo III–IV) [37,40].

Across the included studies, patient populations were heterogeneous. The majority of patients were male, with median age typically ranging between 55 and 70 years [33,37,40]. Tumor laterality was variable, with a slight predominance of right-sided tumors in several cohorts [33].

Tumor size varied widely across studies, reflecting differences in case selection. Histopathological analysis predominantly revealed clear cell renal cell carcinoma, with a high proportion of advanced-stage tumors (≥ pT3) and high-grade disease (ISUP grade 3–4) [4,32,36].

A notable imbalance was observed in thrombus level distribution between surgical approaches. Robotic procedures were more frequently performed in patients with lower-level thrombi (Mayo I–II), whereas open surgery included a higher proportion of patients with advanced thrombus extension (Mayo III–IV) [37,40].

Follow-up duration was heterogeneous and generally shorter in robotic cohorts, often ranging from several months to approximately 2–3 years [34,40].

### 4.2. Reported Perioperative Outcomes

Table 3 summarizes the reported perioperative outcomes across included studies.

#### 4.2.1. Operative Time

Operative time was consistently longer in robotic procedures compared with open surgery. Across the included studies, operative time for robotic surgery ranged from approximately 120 to more than 350 min, whereas open procedures typically ranged from approximately 200 to more than 300 min, depending on thrombus level and surgical complexity [34,37,40]. For instance, Vuong et al. reported a median operative time of 350.5 min for robotic surgery compared with 208 min for open procedures [40].

#### 4.2.2. Estimated Blood Loss

Robot-assisted surgery was consistently associated with lower estimated blood loss (EBL). Reported EBL ranged from approximately 87 to 400 mL in robotic cohorts, compared with 550 to 1850 mL in open surgery [33,37,40]. Similarly, Gu et al. demonstrated significantly lower blood loss in robotic cases (250 vs. 1000 mL), a finding also observed by Beksac et al. (100 vs. 600 mL) [33,37].

#### 4.2.3. Transfusion Rates

Transfusion rates were generally lower in robotic cohorts, ranging from 0% to approximately 6%, compared with up to 50–80% in open surgery [33,37].

#### 4.2.4. Length of Stay

Length of hospital stay was shorter following robotic surgery, typically ranging from 2 to 5 days, compared with 5 to 10 days in open procedures [33,37,40].

### 4.3. Complications

Overall complication rates were comparable between robotic and open approaches [33,37,40]. However, the type and severity of complications differed depending on tumor complexity and surgical approach.

The most commonly reported complications included intraoperative bleeding, postoperative pulmonary complications, thromboembolic events, acute renal failure, and surgical site complications [36,40]. In open surgery, intraoperative complications more frequently involved injury to adjacent organs or major vascular structures, whereas robotic procedures were more commonly associated with technical challenges related to vascular control or the need for conversion to open surgery [19,20,40].

In a comparative study, Vuong et al. reported postoperative complications (Clavien-Dindo grade > II) in 30% of robotic cases and 37% of open procedures [40]. Severe complications (Clavien-Dindo grade ≥ III), including those requiring surgical or radiological intervention, were reported inconsistently but did not demonstrate significant differences between approaches [33,40].

### 4.4. Influence of Tumor Thrombus Level and Tumor Volume

IVCTT level was a major determinant of surgical complexity. Higher-level thrombi (Mayo III–IV) were associated with longer operative time, increased blood loss, higher transfusion rates, and a greater incidence of major complications [4,12,36,42,43].

Renal tumor volume also emerged as an important factor. Baheen et al. showed that larger tumor thrombus volume was associated with increased operative complexity, greater intraoperative blood loss, and a higher likelihood of requiring an open surgical approach [32].

### 4.5. Reported Oncological Outcomes

Oncological outcomes were comparable between robotic and open approaches [38,39]. Median follow-up was variable and generally shorter in robotic cohorts, ranging from approximately 8 to 13 months in comparative studies and up to several years in selected series [34,40].

No consistent differences were observed in OS, CSS, or progression-free survival between surgical approaches. In a large multi-institutional cohort, Sandberg et al. demonstrated no significant differences in OS between surgical approaches, with median OS ranging from 1.5 to 2.5 years [38,39]. Rates of disease progression were similar between groups, occurring in approximately 30–37% of patients in comparative studies [40].

Oncological outcomes were primarily driven by tumor-related factors, including thrombus level, tumor grade, tumor size, and invasion of the IVC wall, rather than the surgical approach [4,36].

### 4.6. Feasibility of Robotic Approach

Robot-assisted IVC thrombectomy was shown to be feasible and safe in carefully selected patients, particularly those with lower-level thrombi (Mayo I–II), limited tumor burden, absence of IVC wall invasion, and procedures performed in high-volume centers [19,20,40].

## 5. Discussion

This systematic review summarizes the current evidence on robot-assisted versus open nephrectomy with inferior vena cava tumor thrombectomy in renal cell carcinoma. Overall, the available data suggest that robot-assisted surgery is feasible in carefully selected patients, particularly those with lower-level tumor thrombi, while open surgery remains essential for more complex cases requiring extensive vascular control, caval reconstruction, liver mobilization, or cardiopulmonary bypass.

### 5.1. Interpretation of Perioperative Outcomes

Across the included studies, robot-assisted surgery was generally associated with lower estimated blood loss, reduced transfusion rates, and shorter hospital stay when compared with open surgery [33,37,40]. These findings are consistent with the potential advantages of minimally invasive surgery, including improved visualization, precise dissection, enhanced instrument dexterity, and the tamponade effect of pneumoperitoneum. However, robotic procedures were often associated with longer operative time, reflecting technical complexity, docking and repositioning requirements, intracorporeal vascular control, and the learning curve associated with robotic IVC thrombectomy [19,20,21,22,23,24,25,26,27,37,40].

### 5.2. Interpretation of Oncological Outcomes

Oncological outcomes were broadly comparable between surgical approaches in the available studies [38,39]. No consistent differences were observed in overall survival, cancer-specific survival, or progression-free survival. These findings suggest that long-term prognosis is primarily determined by tumor-related factors, including thrombus level, tumor size and volume, histological subtype, tumor grade, metastatic status, and invasion of the IVC wall, rather than by the surgical approach alone [4,9,32,36,42,43,44,45,46].

The key oncological objective remains complete resection of the kidney tumor and venous tumor thrombus with adequate vascular control. When these principles are preserved, robot-assisted surgery may offer comparable oncological outcomes in selected patients. However, follow-up in robotic cohorts was generally shorter and sample sizes were smaller than in open series, limiting definitive conclusions regarding long-term cancer-specific outcomes [19,20,21,22,23,24,37,38,39,40].

### 5.3. Patient Selection and Mayo I–II Thrombi

The clinical relevance of the robotic approach appears greatest in patients with lower-level IVCTT, particularly Mayo I–II thrombi. In this subgroup, robot-assisted surgery may be technically feasible and may offer perioperative benefits when performed in experienced high-volume centers [19,20,21,22,23,24,25,26,27,37,40]. These patients usually have more favorable anatomy, limited thrombus extension, and a lower likelihood of requiring complex caval reconstruction.

Nevertheless, a formal pooled subgroup comparison limited to Mayo I–II thrombi was not possible because the included studies reported outcomes heterogeneously and did not consistently provide stratified data according to thrombus level. Therefore, the available evidence supports robotic surgery as an option for carefully selected Mayo I–II cases rather than as a universally applicable alternative to open surgery.

### 5.4. Impact of Selection Bias on Comparative Outcomes

Selection bias remains the most important limitation when comparing robotic and open approaches. In most included studies, the choice of surgical approach was strongly influenced by thrombus level, thrombus complexity, suspected adherence or invasion of the caval wall, and the anticipated need for extensive vascular control or reconstruction [20,21,22,23,24,25,26,35,37,40].

Robotic surgery was typically reserved for patients with lower-level thrombi, limited tumor burden, and favorable anatomical characteristics. In contrast, open surgery was more commonly used for Mayo III–IV thrombi, bulky tumors, complex vascular involvement, and cases requiring liver mobilization or cardiopulmonary bypass [4,9,10,35,44,45]. This imbalance directly affects the interpretation of comparative outcomes. Lower blood loss, reduced transfusion rates, and shorter hospital stay in robotic cohorts should therefore not be interpreted as evidence of superiority over open surgery, but rather as outcomes achieved in a highly selected subgroup of patients.

Although some studies attempted to reduce selection bias through matched analyses or multivariable adjustment, residual confounding remains likely [33,37,40]. Factors such as tumor thrombus volume, caval wall invasion, surgeon experience, center volume, and multidisciplinary support are difficult to fully capture but may substantially influence perioperative and oncological outcomes [4,25,32,35,36,42,43,44,45,46].

### 5.5. Role of Preoperative Systemic Therapy

The increasing use of systemic therapy in RCC may also influence surgical planning. Presurgical or neoadjuvant systemic treatment, including tyrosine kinase inhibitors and immune checkpoint inhibitor-based regimens, may reduce primary tumor size or thrombus extent in selected patients and could potentially affect the feasibility of minimally invasive surgery [1,5,6]. In patients with IVCTT, even partial reduction in thrombus burden may theoretically facilitate vascular control, reduce operative complexity, or influence the choice between robotic and open surgery.

However, the available evidence remains limited, heterogeneous, and largely non-randomized. At present, preoperative systemic therapy should not be considered a standard strategy solely to facilitate robotic IVC thrombectomy. Its role in improving surgical outcomes, downstaging tumor thrombus, or expanding eligibility for minimally invasive surgery requires further prospective evaluation.

### 5.6. Limitations

This systematic review has several limitations. First, most included studies were retrospective, which inherently limits the strength of evidence and introduces potential sources of bias [4,19,20,28,32,33,34,35,36,37,38,39,40]. Second, substantial heterogeneity was observed across studies in patient populations, tumor characteristics, thrombus level distribution, and surgical expertise [4,9,19,20,28,32,33,34,35,36,37,38,39,40]. This variability precluded quantitative synthesis and limits direct comparability of reported outcomes [41].

Third, most studies originated from high-volume centers with significant expertise in complex robotic and vascular surgery [20,21,22,23,24,25,26,27,35,37,40]. As a result, the reported outcomes may not be generalizable to lower-volume institutions or less experienced surgical teams [25,26,27]. Fourth, the geographical distribution of the available evidence was uneven. Most studies originated from North America, Europe, and Asia, whereas data from Africa and South America were absent or markedly underrepresented. variability in reporting standards further complicates interpretation. Differences in the definition and grading of complications, as well as inconsistent reporting of perioperative and oncological outcomes, may affect the reliability of cross-study comparisons [9,29,31,41].

Finally, reporting standards varied between studies. Definitions of estimated blood loss, transfusion, complication grading, follow-up duration, and oncological endpoints were inconsistent [9,29,31,41]. The absence of prospective randomized trials and the relatively short follow-up in robotic cohorts further restrict the ability to draw definitive conclusions regarding long-term oncological equivalence.

### 5.7. Clinical Implications and Future Directions

Robot-assisted nephrectomy with IVC thrombectomy should currently be considered a feasible option for selected patients, particularly those with Mayo I–II thrombi, favorable anatomy, limited tumor burden, and no evidence of extensive caval wall invasion. Such procedures should be performed in experienced centers with expertise in robotic surgery, vascular control, and multidisciplinary perioperative management [19,20,21,22,23,24,25,26,27,37,40].

Open surgery remains the reference approach for complex cases, especially patients with high-level thrombi, extensive vascular involvement, bulky tumor thrombus, suspected caval wall invasion, or anticipated need for major reconstruction. Therefore, robotic surgery should be viewed as a complementary approach for selected cases rather than a replacement for open surgery.

Future research should focus on prospective, multi-institutional studies with clearly defined inclusion criteria, uniform definitions of perioperative and oncological outcomes, and longer follow-up [9,10,29,31,41]. Standardization of reporting, including uniform definitions of perioperative outcomes, complication grading, and oncological endpoints, would facilitate more reliable comparisons across studies [29,31,41].

Studies stratified by Mayo thrombus level, tumor volume, vascular invasion and systemic therapy status are needed to better define which patients benefit most from robotic surgery. The effect of preoperative systemic therapy on tumor thrombus regression, surgical complexity, and approach selection also warrants further investigation.

## 6. Conclusions

Robot-assisted nephrectomy with IVC thrombectomy appears feasible and safe in carefully selected patients and may offer perioperative advantages, particularly in Mayo I–II thrombi managed at experienced centers. However, findings are limited by retrospective study design, heterogeneity, and substantial selection bias. Surgical complexity and prognosis are primarily determined by tumor-related factors, particularly thrombus level and tumor burden. Open surgery remains essential for complex high-level thrombi and cases requiring extensive vascular control. Surgical approach selection should therefore be individualized according to thrombus level, tumor burden, vascular involvement, patient characteristics, and institutional expertise.

## Figures and Tables

**Table 1:** Risk of Bias Assessment Using the Newcastle-Ottawa Scale (NOS).

Study	Selection (Max 4)	Comparability (Max 2)	Outcome (Max 3)	Total Score	Quality Assessment
Abaza et al. [19]	3	0	2	5	Moderate
Abaza et al. [20]	4	0	3	7	High
Baheen et al. [32]	4	1	3	8	High
Beksac et al. [33]	4	2	3	9	High
Blute et al. [4]	4	1	3	8	High
Cheng et al. [34]	3	0	2	5	Moderate
Dell’Oglio et al. [35]	4	2	3	9	High
Durante et al. [28]	3	0	2	5	Moderate
Garg et al. [36]	4	1	3	8	High
Gu et al. [37]	4	2	3	9	High
Sandberg et al./ICORCC [38]	4	2	3	9	High
Sandberg et al. 2025 [39]	4	2	3	9	High
Vuong et al. [40]	4	2	3	9	High

NOS: Newcastle-Ottawa Scale. Quality interpretation: 7–9 points, high quality; 5–6 points, moderate quality; <5 points, low quality.

**Table 2:** Characteristics of included studies.

Key Notes	Mayo Level	Other *n*	Open *n*	Robotic *n*	Total *n*	Study Category	Study Design	Year	Country/Region	Study
Initial robotic experience	I–II	0	0	9	9	Robotic case series	Retrospective	2011	USA	Abaza et al. 2011 [19]
Multi-institutional robotic experience	II–III	0	0	32	32	Multi-institutional robotic series	Retrospective	2016	Multicenter	Abaza et al. [20]
Advanced robotic techniques	II–IV	0	0	5	5	Robotic case series	Retrospective	2025	Europe	Durante et al. [28]
Two-year follow-up robotic series	I–III	0	0	21	21	Single-center robotic series	Retrospective	2024	China	Cheng et al. [34]
Propensity-matched comparison	I–II	0	31	31	62	Comparative cohort	Retrospective matched study	2017	China	Gu et al. [37]
Robot-assisted versus open surgery	I–II	0	30	10	40	Monocenter comparative cohort	Retrospective	2021	France	Vuong et al. [40]
Cytoreductive nephrectomy cohort; included laparoscopic cases	I–IV	25	97	9	131	Comparative cohort	Retrospective	2025	International	Sandberg et al./ICORCC [39]
Open, laparoscopic, and robotic approaches	I–IV	61	308	23	392	Large comparative cohort	Retrospective	2025	International	Sandberg et al. [38]
High-volume center experience	I–IV	0	40	0	40	Open cohort	Retrospective	2024	Europe	Dell’Oglio et al. [35]
Contemporary national cohort	I–IV	0	838	34	872	NCDB cohort	Retrospective database analysis	2019	USA	Beksac et al. [33]
Open radical nephrectomy with IVC thrombectomy	I–IV	0	56	0	56	Survival analysis cohort	Retrospective	2023	India	Garg et al. [36]
Analysis of tumor volume, surgical approach, and complexity	I–IV	0	73	59	132	Tumor volume analysis cohort	Retrospective	2022	China	Baheen et al. [32]
Historical benchmark open series	I–IV	0	540	0	540	Historical open cohort	Retrospective	2004	USA	Blute et al./Mayo Clinic series [4]

Abbreviations: ICORCC, Intercontinental Collaboration on Renal Cell Carcinoma; IVC, inferior vena cava; NCDB, National Cancer Database. Other *n* refers mainly to laparoscopic or other minimally invasive approaches reported in multi-arm comparative studies.

**Table 3:** Perioperative outcomes across included studies.

Key Finding	Complications Reported	Length of Stay (Days)	Transfusion Rate (%)	Estimated Blood Loss (mL)	Operative Time (Min)	Mayo Level	Surgical Approach	Study
Robotic surgery was associated with lower blood loss, fewer transfusions, shorter hospital stay, and fewer major complications in matched Mayo I–II cases.	9.7% vs. 29% major complications	5 vs. 9	6.5 vs. 54.8	250 vs. 1000	150 vs. 230	I–II	Robotic vs. open	Gu et al., [37]
Robotic cases showed lower blood loss and shorter hospital stay in a national database cohort.	No significant difference	4 vs. 6	NR	100 vs. 600	226 vs. 260	I–IV	Robotic vs. open	Beksac et al., [33]
Robotic surgery had longer operative time but lower blood loss and shorter hospital stay.	30% vs. 37% postoperative complications > Clavien-Dindo II	7 vs. 10	50 vs. 63.3	500 vs. 1250	350.5 vs. 208	I–II	Robotic vs. open	Vuong et al., [40]
Minimally invasive approaches were associated with shorter hospital stay in a multi-arm comparative cohort.	NR	9 vs. 6 vs. 4	NR	NR	304 vs. 267 vs. 216	I–IV	Open vs. laparoscopic vs. robotic	Sandberg et al./ICORCC, [39]
Robotic cases had lower transfusion rates, although interpretation is limited by selection bias.	NR	NR	57.7 vs. 0	NR	NR	I–IV	Open vs. robotic	Sandberg et al., [38]
Multi-institutional robotic series demonstrated feasibility in selected patients.	0% major complications	3.2	9	399	292	II–III	Robotic	Abaza et al., [20]
Robotic management was feasible in selected complex cases at experienced centers.	0% major complications	7	0	400	414	II–IV	Robotic	Durante et al., [28]
Single-center robotic experience with acceptable perioperative outcomes.	NR	8.4	NR	~400	NR	I–III	Robotic	Cheng et al., [34]
Open surgery included complex cases across the full thrombus spectrum.	51.7% complications	10.6	NR	1852	303	I–IV	Open	Garg et al., [34]

Values are reported as mean or median where available. NR: not reported.

## Data Availability

No new data were created or analyzed in this study. Data sharing is not applicable to this article.

## Supplementary Materials

The following supporting information can be downloaded at: https://www.mdpi.com/article/10.3390/cancers18142251/s1, Table S1: PRISMA 2020 checklist; Table S2: Database-specific search.
