# Supplementary material for: Robot-Assisted Versus Open Nephrectomy with Inferior Vena Cava Thrombectomy in Renal Cell Carcinoma: Current Evidence and Surgical Trends
Source: Cancers (Basel). 2026 Jul 14;18(14):2251. doi: 10.3390/cancers18142251 (PMC13407142; doi:10.3390/cancers18142251)
Supplement: Supplementary file 1 [file cancers-18-02251-s001.zip › cancers-4372828-supplementary.pdf]

cancers

MDPI

Systematic Review

Robot-Assisted Versus Open Nephrectomy with Inferior Vena Cava Thrombectomy in Renal Cell Carcinoma: Current Evidence and Surgical Trends

Zuzanna Korbecka [1,*], Beata Jabłońska [2] and Robert Król [1]

1 Department of General, Vascular and Transplant Surgery, Medical University of Silesia, St. Francuska 20-24, 40-064 Katowice, Poland; robertk@hot.pl
2 Department of Digestive Tract Surgery, Medical University of Silesia, St. Medyków 14, 40-753 Katowice, Poland; bjablonska@sum.edu.pl
* Correspondence: zuzanna.korbecka@sum.edu.pl

Supplementary Table S1. PRISMA 2020 Checklist

Manuscript: Robot-assisted versus open nephrectomy with inferior vena cava thrombectomy in renal cell carcinoma: current evidence and surgical trends

This completed checklist reports where each PRISMA 2020 item is addressed in the manuscript or supplementary material.

Section	Item	PRISMA 2020 Checklist Item	Location in Manuscript	Comments / Notes
Title	1	Identify the report as a systematic review.	Title page / Title	The title identifies the article as a systematic review of current evidence.
Abstract	2	See the PRISMA 2020 for Abstracts checklist.	Abstract	Structured abstract includes Background/Objectives, Methods, Results, and Conclusions.
Introduction	3	Describe the rationale for the review in the context of existing knowledge.	Introduction	Clinical relevance of RCC with inferior vena cava tumor thrombus and evolving robotic surgery are described.
Introduction	4	Provide an explicit statement of the objective(s) or question(s) the review addresses.	Introduction - final paragraph	The objective is to compare robot-assisted and open nephrectomy with IVC thrombectomy, focusing on perioperative and oncological outcomes and determinants of complexity.
Methods	5	Specify the inclusion and exclusion criteria for the review and how studies were grouped for the syntheses.	Methods - Inclusion and exclusion criteria; Results - Characteristics of included studies	Eligibility criteria and included study types are described.
Methods	6	Specify all databases, registers, websites, organisations, reference lists and other sources searched or consulted, and the date when each source was last searched.	Methods - Search strategy	PubMed, Embase, and Scopus were searched; search limits are described.
Methods	7	Present the full search strategies for all databases, registers and websites, including any filters and limits used.	Methods - Search strategy; Supplementary search strategy file	Search concepts and limits are reported; detailed search strategy may be submitted separately.
Methods	8	Specify the methods used to decide whether a study met the inclusion criteria, including how many reviewers screened each record and report, whether they worked independently, and details of automation tools used.	Methods - Study selection	Study selection process is described. Automation tools were not used for eligibility decisions.
Methods	9	Specify the methods used to collect data from reports, including how many reviewers collected data, whether they worked independently, any processes for obtaining or confirming data from investigators, and details of automation tools used.	Methods - Data extraction	Extracted data items are described. The review used published aggregate data.
Methods	10a	List and define all outcomes for which data were sought. Specify whether all results compatible with each outcome domain were sought.	Methods - Outcomes; Methods - Data extraction	Perioperative parameters, complications, and survival outcomes are defined.
Methods	10b	List and define all other variables for which data were sought. Describe assumptions made about missing or unclear information.	Methods - Data extraction; Results - Characteristics of included studies	Study characteristics, surgical approach, tumor thrombus level, and tumor characteristics were extracted.
Methods	11	Specify the methods used to assess risk of bias in the included studies, including details of tools used and how many reviewers assessed each study.	Methods / Results; Table 3	Risk of bias was assessed using the Newcastle-Ottawa Scale (NOS). Results are presented in Table 3.
Methods	12	Specify for each outcome the effect measure(s) used in the synthesis or presentation of results.	Methods - Data synthesis; Results	Formal pooled effect measures were not calculated because only qualitative synthesis was performed.
Methods	13a	Describe the processes used to decide which studies were eligible for each synthesis.	Methods - Data synthesis; Results	All eligible studies were included in the qualitative synthesis; outcomes are grouped by domain.

Supplementary Table S2. Database-specific search strategies.

Database	Search strategy	Limits/filters
PubMed	(("Carcinoma, Renal Cell"[Mesh] OR "renal cell carcinoma"[Title/Abstract] OR RCC[Title/Abstract] OR "renal cancer"[Title/Abstract] OR "kidney cancer"[Title/Abstract]) AND ("Vena Cava, Inferior"[Mesh] OR "inferior vena cava"[Title/Abstract] OR IVC[Title/Abstract] OR caval[Title/Abstract]) AND ("tumor thrombus"[Title/Abstract] OR "tumour thrombus"[Title/Abstract] OR thrombus[Title/Abstract] OR thrombectomy[Title/Abstract]) AND ("Nephrectomy"[Mesh] OR nephrectomy[Title/Abstract] OR "radical nephrectomy"[Title/Abstract]) AND ("Robotic Surgical Procedures"[Mesh] OR robotic[Title/Abstract] OR "robot-assisted"[Title/Abstract] OR "robot assisted"[Title/Abstract] OR open[Title/Abstract] OR surgery[Title/Abstract]))	English; Humans
Scopus	TITLE-ABS-KEY(("renal cell carcinoma" OR RCC OR "renal cancer" OR "kidney cancer") AND ("inferior vena cava" OR IVC OR caval) AND ("tumor thrombus" OR "tumour thrombus" OR thrombus OR thrombectomy) AND (nephrectomy OR "radical nephrectomy") AND (robotic OR "robot-assisted" OR "robot assisted" OR open OR surgery))	English
Web of Science	TS=(("renal cell carcinoma" OR RCC OR "renal cancer" OR "kidney cancer") AND ("inferior vena cava" OR IVC OR caval) AND ("tumor thrombus" OR "tumour thrombus" OR thrombus OR thrombectomy) AND (nephrectomy OR "radical nephrectomy") AND (robotic OR "robot-assisted" OR "robot assisted" OR open OR surgery))	English
Cochrane Library	("renal cell carcinoma" OR RCC OR "renal cancer" OR "kidney cancer") AND ("inferior vena cava" OR IVC OR caval) AND ("tumor thrombus" OR "tumour thrombus" OR thrombus OR thrombectomy) AND (nephrectomy OR "radical nephrectomy") AND (robotic OR "robot-assisted" OR "robot assisted" OR open OR surgery)	English, where available
